# The Impact of Rape Myth Education on Jury Decision-Making: A Systematic Review

**DOI:** 10.1177/15248380241270082

**Published:** 2024-09-13

**Authors:** Lara Flynn Hudspith, Nadia Wager, Dominic Willmott, Bernard Gallagher

**Affiliations:** 1 The University of British Columbia, Vancouver, Canada; 2 Teesside University, Middlesbrough, UK; 3 Loughborough University, UK; 4 SWPS University, Wroclaw, Poland; 5 University of Central Lancashire, Preston, UK

**Keywords:** sexual assault, intervention, jury decision-making

## Abstract

A systematic review of research exploring the impact of providing rape myth countering information to mock-jurors was conducted. The primary aim of the review was to inform the development of an educational intervention for jurors to reduce potential bias in their decision-making based on belief in prevalent rape myths. In total, the following 12 databases were searched: British Education Index, CINAHL, Child Development and Adolescent Studies, Criminal Justice Abstracts, Educational Administration Abstracts, ERIC, MEDLINE, PsycArticles, PsycInfo, PubMed, Scopus, and Social Care Online. The databases were filtered to return peer-reviewed publications, written in English, and published between 1980 and 2023. The search returned 6,119 potential articles. After duplicates were removed, the 3,202 remaining publications were screened. Only studies that presented rape myth countering information to participants within a mock-juror paradigm were included. Studies that did not compare an information condition to a no-information control condition were excluded, as were those which concerned male rape myths, given the focus on female rape myth beliefs in this review. Eight studies were reviewed and were critically appraised according to validity criteria utilized by Dinos et al. Some evidence emerged to suggest that the provision of judicial directions, expert witness testimony, and complainant statements regarding rape myths can impact upon jury decision-making although the findings were mixed overall. Study limitations regarding internal and external validity were frequently present. Recommendations regarding future research questions and methods are advanced, including those that can inform the development of an effective intervention for jurors.

## Introduction

Conviction rates for rape remain low in many countries (Daly & Bouhours, 2010). For example, in England and Wales the rape conviction rate fell to an all-time low in 2020, with only 1,439 (2.6%) of reported rapes resulting in convictions (Topping & Barr, 2020), and from 2020 to 2021, there was a 2.4%-point reduction in conviction rates. While there are several factors that contribute toward low conviction rates, it is important to recognize the significant role that juror bias can have on jury decision-making, and consequently on case outcomes and conviction rates. A wealth of research has shown that juror decisions can be affected by various forms of bias, including the attitudes that jurors hold (e.g., attitudes toward a particular crime or racist attitudes) or their personality traits (e.g., authoritarianism; Willmott, 2017). With regards to rape cases, it is well documented that attitudes toward rape in particular, namely belief in “rape myths” (RMs), can impact upon jury decision-making (JDM; Leverick, 2020; Willmott & Hudspith, 2024).

The concept of RMs was first developed in the 1970s (Brownmiller, 1975) and was then formally defined in 1980 as “prejudicial, stereotyped, or false beliefs about rape, rape victims and rapists” (Burt, 1980, p. 217). Since then, others have incorporated the functions of RMs into proposed definitions, for example, Lonsway and Fitzgerald (1994) define RMs as “attitudes and beliefs that are generally false, but are widely and persistently held, that serve to deny and justify male sexual aggression against women” (p. 134).

RMs may inform individuals’ perceptions of rape through a phenomenon known as psychological scripts or interpretive schemas (Willmott et al., 2021). Such RM-informed schemas or scripts may lead individuals to believe in narrow conceptions of what constitutes rape—conceptions that diverge from legal definitions (Ryan, 2011). For instance, individuals may believe in the “real rape” myth, endorsing the view that legitimate rape occurs only outdoors with a stranger attacking a victim who physically resists and sustains injuries (Estrich, 1987). Belief in such erroneous rape scripts fosters an environment where acquaintance or partner rape is normalized and sexual violence is justified and sustained (Edwards et al., 2011).

Belief in RMs is widespread across society (Amnesty International UK, 2005; Hudspith et al., 2023). As such, there is the potential for the progression of cases through the criminal justice system (CJS) to be impacted by RMs as such beliefs might affect the decisions of victim-survivors and professionals within the system, ranging from police officers, those working within the Crown Prosecution Service, barristers, to judges, and others (Temkin & Krahé, 2008; Willmott & Hudspith, 2024).

With regards to victim-survivors, research has shown that individuals might be reluctant to report their experiences to the police based on RMs; victim-survivors’ own RM beliefs might prevent them from reporting as they may not label their experience of rape (Lilley et al., 2023a; Lorenz et al., 2019), and they also might be influenced by the beliefs that they anticipate officers to have, namely they might be fearful of being blamed or disbelieved by police officers who endorse RMs (Daly & Bouhours, 2010; Murphy-Oikonen et al., 2022; Stewart et al., 2024).

RMs can also influence police and prosecutors’ actions and decisions. Their personal endorsement of RMs might directly influence their decisions, while their anticipation of the beliefs of other CJS decision-makers may have indirect impacts (Daly & Bouhours, 2010; Davies et al., 2022; Gekoski et al., 2023; Hohl & Stanko, 2015; Jordan & Mossman, 2019; Murphy & Hine, 2019; Salerno-Ferraro & Jung, 2022).

Furthermore, RMs can also have an influence upon cases that are progressed to trial. Court observation studies have consistently shown that RMs are introduced by judges and by both prosecution and defense barristers to challenge complainants’ credibility, suggest they consented, or to suggest they are to blame for being assaulted (Burgin, 2019; Burgin & Flynn, 2019; Daly, 2022; Smith & Skinner, 2012; Temkin et al., 2018; Quilter et al., 2022, 2023; Zydervelt et al., 2017). Importantly, such use of RMs might impact upon JDM. In fact, mock-trial research has consistently shown that judgments of credibility, blame, and guilt may be influenced by expectations based on RMs. For example, level of complainant distress has been shown to impact upon credibility ratings (Nitschke et al., 2019), and complainant’s “respectability,” attractiveness, clothing, and intoxication level at the time of the assault have been shown to impact upon attributions of blame, as has the relationship between the complainant and the defendant (Gravelin et al., 2019; Pollard, 1992; Whatley, 1996). Beyond this, mock-juror research has also found that those who score highly on RM scales, known as high Rape Myth Acceptance (RMA), are not only more likely to blame complainants and attribute higher levels of responsibility to them (Gravelin et al., 2019; Maeder et al., 2015; Romero-Sánchez et al., 2012; Stevens et al., 2024; Süssenbach et al., 2013; Vrij & Firmin, 2001; Zidenberg et al., 2019), but they are also less likely to reach a guilty verdict (Dinos et al., 2015; Leverick, 2020; Lilley et al., 2023b).

Qualitative analysis of mock-jury deliberations has also shown that RM beliefs can arise during discussions, and that they may be endorsed by jurors irrespective of their individual RMA scores captured quantitatively pre-trial (Leverick, 2020). Several dominant RMs have been identified across such studies. These include the beliefs that genuine victims would verbally resist throughout an assault (i.e., scream and shout), physically resist throughout, such that they sustain injuries afterward, including internal injuries, report the assault to the police immediately, not maintain a relationship with the alleged perpetrator, and present as distressed while testifying; false allegations are common; and rape is a crime of passion based on uncontrollable urges (Willmott et al., 2021).

Recent research conducted using data from genuine court cases further indicates that RMs may influence juror decision-making. For instance, Lundrigan et al. (2019) examined 394 stranger rape cases to determine the factors that predicted case outcomes. Cases involving an outdoor location were 50% more likely to result in a conviction than cases reported to have occurred indoors. The authors noted that this finding may reflect the influence that the “real rape” myth can have on jurors (Estrich, 1987).

Thomas (2020) conducted a post-trial survey with genuine trial jurors in England and Wales and, in contrast to the conclusions of a considerable body of prior research, concluded that “hardly any” jurors believe in RMs (p. 12). Although this research is valuable, given the rare insight it provides from genuine jurors, it is recognized that this study was bound by particular constraints regarding the scope and nature of the testing that could be conducted (see Daly et al., 2023 for a detailed review). Crucially, Thomas was neither able to directly test whether jurors’ RMA was associated with their verdicts, nor was she able to explore the impact that individuals with high RMA may have had on those with low RMA during deliberations. Further, the internal validity of the study, rather than that of a RMA scale, was weak as discrete isolated RMs were selected for the study (Willmott et al., 2021).

The wealth of mock-trial research that has demonstrated that RMA can impact upon JDM should not be dismissed based on Thomas’ (2020) conclusion, despite this conclusion being based upon research conducted with genuine jurors. There is a high likelihood that members of a jury might believe in RMs given the widespread acceptance of such views across society (Amnesty International UK, 2005). Indeed, while Thomas (2020) stated that “hardly any” jurors believed in RMs, she reported findings that in fact show a substantial minority of jurors either believed in several RMs or were uncertain about their veracity. For example, over 35% of jurors surveyed were either unsure whether perpetrators were more likely to be strangers or acquaintances, or believed that perpetrators were more likely to be strangers. Furthermore, results of Tinsley et al.’s (2021) study, which was also conducted with genuine jurors, found evidence that jurors held misconceptions about sexual violence that ultimately impacted upon their decision-making and final verdicts.

Given the wealth of prior research from varied disciplinary and methodological perspectives which finds evidence of a prejudicial effect of RMs on JDM, several proposals have been put forward as to how this apparent issue could be addressed. Some authorities have proposed that jurors should be required to justify their decisions in rape cases (Auld, 2001). While Krahé et al.’s (2007) research supports this proposal, they noted that further research exploring the effects of this approach was required before it could be formally considered.

Others have proposed that individuals should be screened such that those who hold belief in RMs are excluded from service (Willmott et al., 2021), though it is unclear how such a system would operate and what effect it might have (Krahé & Temkin, 2013). Moreover, some authorities have contested the value of screening jurors based on scores on abstract RM questionnaires, arguing that regardless of how individual jurors score, it is the introduction and use of rape mythology within an applied case-specific context (i.e., during group deliberation) whereby RMs become influential (Munro, 2019). Further, questionnaire responses may arguably be affected by social desirability bias; individuals might give answers in line with what they believe to be socially acceptable, rather than providing truthful answers (Hans & Jehle, 2003).

Researchers have also considered the use of judge-only trials (Dripps, 2009; Finn et al., 2011; Krahé & Temkin, 2013). Retter (2018) noted that while this approach might increase the transparency of the process, judges are not necessarily more adept at making non-biased judgments on such cases compared to jurors. Judges too may endorse RMs; RMs that could affect their decision-making (Embury-Dennis, 2018). Further, without group deliberation, it would not be possible for biases to be challenged, with the result that only one individual’s reasoning would be applied to a case.

Others have proposed that expert witnesses be called upon in rape trials. For instance, in 2006, the UK government held a consultation regarding prosecution barristers introducing general expert witness evidence (Office for Criminal Justice Reform, 2006)—testimony regarding information about common behaviors associated with, and psychological reactions to, rape. Case-specific information is unlikely to be necessary to inform jurors’ assessments of the complainants’ credibility, beyond that of their existing knowledge. However, a key objection against the introduction of this type of testimony is its potential to usurp the jury’s role in assessing credibility. Ward (2009) argued that jurors might over-rely on such testimony, treating experts as the authority on the issues discussed, rather than engaging in decision-making based on the case-specific evidence. Further concerns related to the possibility of general expert testimony “expanding” into case-specific territory, and the possibility of prosecution experts to be challenged by defense experts leading to a time-consuming and expensive “battle of experts,” ultimately prevented the implementation of the proposal (Ward, 2009).

One approach that has been implemented in England and Wales, and elsewhere, is the provision of judicial directions to jurors about RMs. Judge’s directions are mandatory in some jurisdictions, whereas in others, including England and Wales, the provision is optional, albeit frequently used (Judicial College, 2020). The scope for further development of directions in England and Wales has been noted. For example, Ellison (2019) argued that directions are limited as they do not include contextual information, such as the preexisting relationship between the parties (e.g., when the defendant is an (ex)intimate partner), and Temkin et al. (2018) noted that directions that cover responses to marital rape need to be incorporated.

Given the lack of standardization of judicial directions in some jurisdictions, a further limitation of this approach is that they are inconsistently utilized across places and cases (Temkin, 2010; Temkin et al., 2018; Thomas, 2020). To counter this in the United Kingdom, it has been proposed recently that jurors should receive standardized information to counter RMs, and this is reportedly being piloted in some courts (Gillen, 2019; HM Government, 2021; Law Commission, 2023).

## Review Aims

Having reviewed a range of empirical studies that utilized different research methods and designs, it was concluded that there is overwhelming evidence to suggest that RMA may impact upon JDM. Given this conclusion, it is deemed valuable to conduct a systematic review of research examining the impact of implementing RM interventions that aim to dispel jurors’ belief in RMs, and thereby reduce the impact that RMs have on JDM, via the presentation of RM-countering information. RM interventions include those outlined above (i.e., the presentation of RM-countering information via judicial directions, or via expert witnesses), as well as others, such as the presentation of RM-countering information via prosecution statements or complainant statements. The aim of the review is to explore the effectiveness of this range of RM interventions to ultimately determine how this issue might be addressed. While Hudspith et al. (2023) reviewed research that had assessed the impact of providing RMA interventions to various populations across a number of different settings, the focus of the current review was on research exploring the impact of providing RM interventions specifically to “jurors.” Due to the legislative restrictions that preclude direct assessment of the impact of RM-debunking information on genuine jurors’ decision-making, the review examined this issue in the context of mock-jury studies that used simulated rape trial scenarios. The aims of the review were to examine the effectiveness of interventions implemented with mock-jurors; critically appraise the evidence base; and provide policy and research recommendations.

Leverick’s (2020) thematic review of research that assessed juror RM education concluded that such information should be provided to jurors. The current systematic review builds on this work in two key ways. First, the current review is systematic rather than thematic. Indeed, to complete the present review, a systematic search and review of all relevant evidence based upon predefined inclusion criteria was conducted, such that it was less likely that relevant research would have been omitted from this review. Second, not only did the current review examine research assessing expert witness testimony and judicial instructions, it also included research exploring the impact of prosecution and complainant statements.

## Method

A systematic review of research assessing the impact of presenting RM interventions to mock-jurors within a mock-trial paradigm was conducted.

### Search Strategy

Search strings were developed to capture articles that assessed the impact of providing RM-debunking information to participants in mock-trials. Given that several titles and abstracts of relevant articles did not make reference to providing a RM intervention specifically, despite providing RM-debunking information to participants, numerous general terms such as “address*” were included to ensure all relevant results were captured.

The following is an example of the terms used to search titles, abstracts, and keywords, adapted as necessary across databases: “((rape AND myth) OR (rape AND belief) OR (rape AND view) OR (rape AND attitude) OR (rape AND misconception) OR “rape supportive” OR “rape accepting”)) AND (program* OR intervention OR address* OR reduc* OR educa* OR chang* OR debunk* OR prevent* OR lower* OR decreas* OR (mock AND trial) OR (trial AND simulation) OR (mock AND jury) OR (mock AND juror)).”

Twelve electronic databases were searched (British Education Index, Child Development and Adolescent Studies, CINAHL, Criminal Justice Abstracts, Educational Administration Abstracts, ERIC, MEDLINE, PsycArticles, PsycInfo, PubMed, Scopus, and Social Care Online), filtered to return only peer-reviewed journals that were published in English between November 1980 (date of publication of Burt, 1980) to July 2023.

### Screening Process

**Figure 1.**
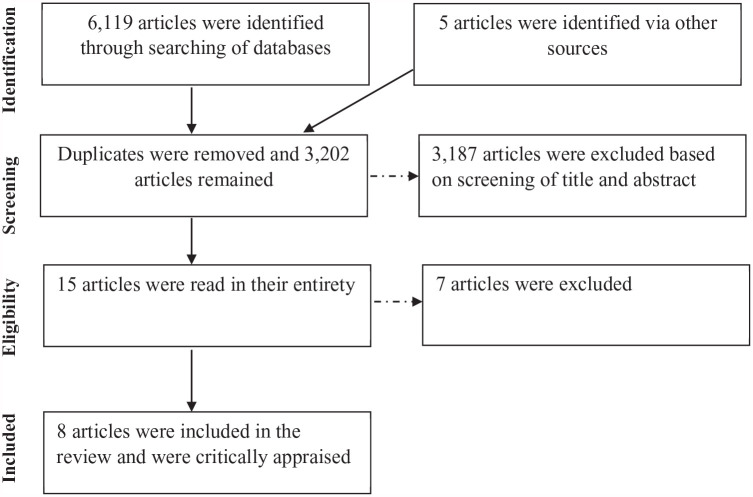
A flowchart to show the conducted screening process.

Duplicates were removed and the first author screened the remaining articles based on the titles and abstracts. Following this, the articles were screened again by the first author based on the full text. A sample of the included and excluded articles were then read by two of the co-authors (NW and DW). Additional articles that were referenced within the included articles and appeared potentially relevant were also screened (Figure 1).

#### Inclusion and Exclusion Criteria

Articles were deemed relevant to be included if they reported on research that assessed the impact of presenting RM-debunking information to mock-jurors within a mock-juror paradigm. They were considered to have used such a paradigm if they had presented a rape case to participants in any format, such as via a live re-enactment or an audiotape. If the research design included the presentation of some form of information to jurors that aimed to challenge RMs (i.e., a RM intervention) and compared this to a no-information control condition, the article was included within the review. Both qualitative and quantitative research was included within the review. Research focusing on RMs attributed to male victims were excluded as RMs regarding adult females were the focus of the review. The rationale for focusing upon female RMs was that while it is acknowledged that males also experience sexual violence, rape is a gendered crime such that the vast majority of allegations, investigations, prosecutions, and jury trials relate to cases involving female complainants and male defendants (Willmott et al., 2021).

### Data Extraction

Data was extracted from the included articles and collated into several tables. Data regarding the RM intervention presented and the key findings of studies are given in Table 1. Once this data was collated, the characteristics of the research process detailed in the articles were critically appraised. The characteristics of interest were split into those relating to internal validity and external validity (Tables 2 and 3). This was based on the critical appraisal process detailed in Dinos et al. (2015), which was adapted to suit the context of this review.

**Table 1.** RM Information Presented and the Research Findings.

Author	Intervention	Study Conditions	Intervention Content	Theory	Key Findings
Gray (2006)	Judicial directions	1. Pro RM 2. Anti-RM 3. Neutral control	1. False allegations are sometimes made 2. False allegations are unlikely 3. Compare each account against the other	None	1 were more likely than 2 to be confident that the defendant was innocent.
Ellison and Munro (2009)	Expert testimony Judicial directions	1. Expert 2. Judicial directions 3. Control	1 and 2. General empirically supported information regarding varied responses to rape (i.e., regarding reporting, demeanor when giving evidence, and physical responses during rape)	None	Those who received information regarding reporting or demeanor rated this as important/helpful and their deliberations differed to those who did not. Information regarding physical responses to rape did not affect deliberations. A larger percentage of 3 than 1 and 2 felt victim distress and a non-delayed report would affect decisions. Most felt that victim injury would affect decisions.
Brekke and Borgida (1988) 1	Expert testimony	1. Standard first 2. Specific first 3. Standard last 4. Specific last 5. Control	1. False allegations are rare, rape is under-reported, most are committed by acquaintances, it is a crime of violence not passion and women may not resist due to fear of additional violence 2-1. with discussion of a hypothetical scenario	Base rate fallacy	2 and 4 were more likely than 1 and 3 to give a guilty verdict, give harsher sentences, and consider the complaint as credible. They were less likely to think the complainant consented and viewed the defendant as less likeable and credible.
Brekke and Borgida (1988) 2	Expert testimony	1. Standard first 2. Specific first 3. Length control to 2 4. Concrete control	As in Study 1	None	2 and 4 saw the victim as more credible, more moral, less responsible, and less likely to have consented than 1. They also saw the defendant as more responsible and less credible than 1, and gave more guilty verdicts.
Brekke and Borgida (1988) 3	Expert testimony	1. Standard first 2. Specific first 3. Standard last 4. Specific last 5. Control	As in Study 1	None	2 were more likely than others to discuss the helpfulness of the witness. They discussed resistance for <2% of the deliberations. This was dominant in other groups’ deliberations.
Spanos et al. (1991)	Expert testimony	1. Crown’s case only 2. Crown’s case and defendant’s testimony 3. Both 2 and expert statement 4. Both 3 and cross-examination of expert	Information about Rape Trauma Syndrome and common, erroneous assumptions about rape	None	Pre-deliberation individual ratings of guilt and verdicts did not differ across conditions. Those in 3 were more likely to reach a unanimous guilty verdict than those 1 and 2, but those in 4 were less likely than 3 to do so. Those in 3 were also more likely to reach an individual post-deliberation guilty verdict than those in 4. Those in 3 and 4 were more likely to believe the complainant than those who saw trial evidence without the expert statement.
Klement et al. (2019) 1	Expert testimony	1. Defense expert 2. Prosecution expert 3. Control	1. 50–90% of allegations are false 2. Only 2%–10% of allegations are false	None	Outcomes not moderated by RM information.
Klement et al. (2019) 2	Information from barristers	1. Defense statement 2. Prosecution statement 3. Control	1. Arguments made that the case did not reflect a typical rape scenario 2. Statement that there is no typical rape scenario	None	Outcomes not moderated by RM information.
Ryan and Westera (2018)	Expert testimony Complainant statement	1. Expert 2. Complainant statement 3. Both 1 and 2 4. Control	1. Freezing is a common response to rape 2. Complainant’s explanation as to why she froze (fearful that the defendant would kill her)	Story Model (Pennington & Hastie, 1992)	There were no main effects or interactions of 1, 2, and 3 on guilt or complainant credibility or blameworthiness. 1 rated the defendant as less to blame than 4. 3 rated the defendant as more to blame than 2. 3 discussed the legal definition of rape more than 4 and 2. 1 gave more evidence-based reasons for verdicts than 2.
Pang et al. (2022)	Standard judicial direction Rape myth education via the judge	1. Standard direction control 2. Both 1 and rape myth information	Caution against three common rape myths and statistics about them	None	Groups did not differ on quantitative measures. 2 scrutinized the complainant less than 1. 2 placed scrutinized the defendant more than the complainant, and did so more than 1. 2 resisted RMs.1 adhered to RMs. 2 were confident in their guilty verdict. 1 questioned verdict.
Nitschke et al. (2023)	Judicial directions	1.Trauma education 2.Standard witness instruction 3. Control	1. Explanation as to why complainants might not show emotions 2. Advisory for jurors to think carefully about the evidence to evaluate the complainant	Heuristic processing	Only 3 evaluated an emotional complainant as more credible than an unemotional complainant. 3 thought the defendant was more likely to be guilty than 2. 1 and 2 rated the complainant as less credible than 3.

RM = rape myths

**Table 2.** Data Extracted Regarding the Internal Validity of the Studies.

Author	Sample	Power Analysis	Condition Allocation	Validity/Reliability of Other Measures	Baseline RMA	Other Confounds
Gray (2006)	180 students	No	Not described	Constructed for the study	Not baseline, but high and low RMA participants compared	None identified
Ellison and Munro (2009)	233 jury-eligible members of the public	No	Not described	Constructed for the study	Not measured	None identified
Brekke and Borgida (1988) 1	208 students	No	At the jury-level	Constructed for the study	Not measured	None identified
Brekke and Borgida (1988) 2	144 students	No	Not described	Constructed for the study	Not measured	Length of trial /information presented & recall of info
Brekke and Borgida (1988) 3	208 students	No	Not described	N/A – Qualitative analyses	Not measured	Not identified
Spanos et al. (1991)	219 students	No	Random	Not described	Not measured	ATWS—at baseline
Klement, et al. (2019) 1	97 jury-eligible students	No	Random	Measure previously used and good reliability reported	Groups not compared	None identified
Klement et al. (2019) 2	83 jury-eligible students	No	Random	Measure previously used, but reliability not described	Groups not compared	None identified
Ryan and Westera (2018)	280 jury-eligible students	Yes	Not described	Constructed for the study	Groups not compared, but RMAS score used as co-variate	None identified
Pang, Davies and Liu (2022)	24 eligible jurors	No	Participant availability	Constructed for the study	Not measured	None identified
Nitschke et al. (2023)	1,085 students and community members	Yes	Random	Constructed for the study	Not measured	Length of the intervention materials

ATWS = Attitudes Toward Women Scale; RMAS = Rape Myth Acceptance Scale.

**Table 3.** Data Extracted Regarding the External Validity of the Studies.

Author	Population	Trial Type	Case Material	Judgment Type Regarding Guilt
Gray (2006)	Public	Written	Unclear	Individual judgment of guilt
Ellison and Munro (2009)	Students	Live re-constructions	Informed by experts	Group deliberation and post-deliberation questionnaires (verdicts given but not reported)
Brekke and Borgida (1988) 1	Students	Audiotape	Based on a real case	Pre-deliberation individual verdict, post-deliberation group verdict, and post-group deliberation questionnaire (including an individual verdict)
Brekke and Borgida (1988) 2	Students	Audiotape	As in study 1	As in study 1
Brekke and Borgida (1988) 3	Students	Audiotape	As in study 1	Group deliberations
Spanos et al. (1991)	Students	Video and audiotape	Unclear	Individual judgment of guilt (rating) and verdict and post-deliberation group verdict
Klement et al. (2019) 1	Students	Written	Unclear	Individual judgment of guilt
Klement et al. (2019) 2	Students	Written	Unclear	As in study 1
Ryan and Westera (2018)	Students	Written	Unclear	Independent judgment of guilt
Pang et al. (2022)	Public	Written and video	Based on a real case	Post-deliberation group verdict
Nitschke et al. (2023)	Public and students	Written	Unclear—appears to be produced for the study	Individual judgment of guilt

## Findings

### Research Findings

Eight articles were reviewed. Five of the eight articles reported on studies exploring the impact of expert witness testimony. Of these five, three articles also reported on the impact of other methods of conveying RM information to mock-jurors, in addition to expert witness testimony. These were barrister statements, complainant statements, and judicial instructions. The remaining three studies explored the impact of judicial instructions only.

As two of the articles reported on more than one study, a total of eleven studies were reviewed. Three of the eleven studies were conducted in England, five in the US, one in Canada, and one in Australia. One study, which was conducted online, recruited participants from both England and Australia. The total number of participants across the 11 studies was 2,542. Participant ages were only reported regarding the samples recruited within five of the eleven studies. The ages ranged from 18 to 70 across these five studies. All but one (woman only) sample consisted of both men and women; however, the split between men and women was not always equal. Further, it was unclear whether participants were restricted to recording their gender as either “male” or “female” in several studies, thus participant’s gender identities might not have been represented and recorded. The ethnicity of participants was often not reported. Overall, it is not possible to provide a conclusive statement as to the diversity of the samples in the reviewed studies. However, it is a strength of the review that the included studies consisted of samples recruited from a range of countries, and that large age ranges were reported in four of the five studies that provided information as to participants’ ages.

Gray (2006), Ellison and Munro (2009), Pang et al. (2022), and Nitschke et al. (2023) reported that the provision of judicial instructions had an impact on JDM. Gray (2006) found that those who received instructions supportive of RMs were more likely to be confident that the defendant was innocent than those who received anti-RM instructions. Similarly, Ellison and Munro (2009) found that deliberations differed across conditions; those who received judicial instructions regarding delayed reporting and complainant’s demeanor in court were less likely to consider such factors as relevant throughout deliberations and made fewer references to such issues. In addition, participants who received such directions were less likely than those who did not to agree that an immediate report to the police or signs of the complainant’s distress would have affected their decisions. However, the direction given regarding physical resistance did not appear to impact upon decision-making. Ellison and Munro (2009) argued that although the jurors understood this information, they did not see it as connected to the acquaintance rape presented as they perceived tonic immobility as related only to stranger rape experiences.

Pang et al. (2022) reported mixed results regarding the impact of judicial directions. Although they found no differences between groups in relation to the quantitative measures they employed, they did report differences between the groups’ deliberations. The authors state that those in the intervention group placed less scrutiny on the complainant than did the control group. They further stated that the intervention group placed more scrutiny on the defendant than the complainant, and that they did so more than the control group. The content of the judicial directions given to participants was not specified. However, examples of the RMs that were discussed in the deliberations were provided. Pang et al. (2022) reported that those in the intervention group demonstrated an awareness of, and resistance to, RMs (e.g., RMs regarding consent) whereas those in the control group demonstrated an adherence to RMs (e.g., that men do not intend to sexual assault others, rather they get “carried away” due to natural “sexual urges”). They also reported that those in the intervention group were confident in stating that their verdict was guilty whereas the control group questioned their decision and appeared less confident.

Nitschke et al. (2023) reported differences between the control group and the two groups who received judicial instructions with regards to participants’ perceptions of complainant credibility and defendant guilt. Those in the intervention groups were less likely than those in the control group to be influenced by the biased belief that there is a link between the level of emotion expressed by a complainant and their credibility. Indeed, those who received no intervention found an emotional complainant to be more credible than an unemotional complainant, whereas those in the intervention groups reported no difference in their perception of the credibility of emotional versus unemotional complainants. While those who had received an intervention were less influenced by this stereotype, they appeared to be overall more skeptical of the complainants, as compared to those in the control group, finding complainants to be less credible than did the control group. Nitschke et al. (2023) also found that participants in the control condition perceived the defendant as more likely to be guilty, compared to individuals in the standard instruction intervention group.

Ellison and Munro (2009) explored the use of judicial directions as well as examined the impact of expert witness testimony, reporting that such testimony was equally effective in addressing participants’ beliefs regarding demeanor and reporting. This finding concurs with Spanos et al.’s (1991) and Brekke and Borgida’s (1988) findings that expert witness testimony influences decision-making. However, Spanos et al. (1991) reported that the effect of expert witness testimony was reversed when an expert was cross-examined. In addition, Brekke and Borgida (1988) argued that jurors use the information presented to them only if it was explicitly linked to the case, and the connection between the information and the case was clarified. Ellison and Munro (2009) dispute this as they found that presenting general information without explicitly stating its connection to the case still had an impact on decision-making. Although they reported that both judicial directions and expert testimony regarding freezing responses to rape were ineffective, due to participants not seeing the link between such information and the case at hand, they argued that further explanation of this point, rather than case explicitness, would have increased its effectiveness.

Ryan and Westera (2018) reported that the presentation of an expert witness led to lower ratings of defendant blame than when such testimony was not presented. However, when expert testimony was combined with a complainant statement, ratings of defendant blame increased. The authors posited that the expert statement presenting tonic immobility as a common response to rape was seen as relevant to the case only when participants heard a complainant explaining *why* they personally froze.

Ryan and Westera (2018) also reported that the combination of expert testimony and complainant statements was associated with participants making more comments regarding legal tests during deliberations than participants who received the complainant statement on its own or neither of the statements. Nevertheless, they reported no significant impact of expert testimony on perceptions of complainants’ credibility or blame, or defendant guilt. Similarly, Klement et al. (2019) also found that expert witness testimony had no impact on mock-jurors’ views of the case presented.

Klement et al. (2019) examined the impact of defense and prosecution barristers presenting RM-debunking or RM-supportive information. They found that provision of such information from barristers did not impact on mock-jurors’ views.

#### Methodological Factors

The internal validity of the studies was often compromised. Sample sizes in each study, other than Pang et al.’s (2022), did not appear to be particularly low; however, a priori power analysis was reported in only two studies (Nitschke et al., 2023; Ryan & Westera, 2018). Hence, the studies may not have been sufficiently powered to attain statistical significance for any observed effects. In addition, the methods by which participants were allocated to conditions was rarely described. Thus, it is unclear whether random allocation occurred (Brekke & Borgida, 1988; Ellison & Munro, 2009; Gray, 2006; Ryan & Westera, 2018). Randomization ensures that key variables that have the potential to confound results are equally distributed across groups. This issue is important given that potential confounding variables were rarely measured, and it is unclear whether baseline scores on key measures were similar across experimental conditions. For example, though RMA was measured in several studies, this was to determine the relationships between RMA and outcome variables, rather than to assess whether RMA levels were equivalent across groups at baseline. Assessing whether RMA levels of each group were similar at baseline would have been beneficial as this would have permitted statistical controlling for any lack of baseline similarity.

Only two studies discussed potential confounding variables. Spanos et al. (1991) administered the Attitudes Toward Women Scale (ATWS; Spence et al., 1973). They found that ATWS scores were similar across groups at baseline and therefore did not have to be controlled for in the analyses. Brekke and Borgida (1988) considered the length and recall of the RM information presented. No effects of such variables were found. Although neither of these studies found effects of the possible confounding variables, the value of ensuring that confounds are considered is not undermined.

There were also issues with the measures used to assess participants’ case judgments (e.g., verdict, victim blame). The validity and the reliability of such measures were typically unknown, as the measures were often created by study authors.

Although beyond the scope of the current review’s aims, it is worth nothing that the RMA measures employed within the studies have been criticized. The validity of the Rape Myth Acceptance Scale (RMAS; Burt, 1980) has been questioned due to the poor wording of some items, which might have led to these items being variously interpreted by different participants. Thus, concerns have been raised that the same responses from different participants may in fact reflect different beliefs. Furthermore, the RMAS has been criticized as two of the items assess knowledge of statistics of sexual violence, rather than attitudes, and the scale focuses on victims, failing to address other relevant matters (Payne et al., 1999). It is recommended that the Acceptance of Modern Myths about Sexual Aggression Scale (Gerger et al., 2007) should be used in future studies. One could argue that this is particularly pertinent with regards to future mock-juror research with non-student samples given that other measures may use language specific to US university students, limiting their use within community populations (Schlegel & Courtois, 2019).

There are also several external validity issues. First, although most articles stated that the sample consisted of individuals who were eligible jurors, only three of the eleven studies recruited from the general public rather than student populations (Ellison & Munro, 2009; Nitschke et al., 2023; Pang et al., 2022). This is arguably problematic as, while studies tend to demonstrate that the verdicts given by student samples and community samples are comparable (Bornstein et al., 2017), individuals who are more educated tend to exhibit lower RMA than those who have undertaken a lower level of education (Suarez & Gadalla, 2010).

Second, the format of the mock-trials was often unrealistic. Only one of the studies presented a live trial re-enactment (Ellison & Munro, 2009), whereas the others presented written or audio materials. Similarly, only two studies scripted a case using the details of a real case (Brekke & Borgida, 1988; Nitschke et al., 2023). Research findings may not be replicated in real trials where the complexity, quantity, and format of the information presented differs.

Finally, the mock-trials were limited with regards to the judgment participants were required to make. Often, participants did not deliberate, rather only individual judgments and verdicts were assessed. This is problematic as omitting group deliberations represents a departure from genuine trial procedures, thereby reducing the realism of procedures, and recording only individual verdicts precludes the examination of changes to verdicts post-deliberation.

#### Conclusions and Recommendations

Although the findings were inconsistent across the reviewed studies, there was some evidence to suggest that providing information that challenges RMs can reduce their influence on jurors’ decision-making (see Table 4). Furthermore, several recommendations can be made regarding how to enhance the effectiveness of various approaches in reducing the bias created by adhering to RMs, and with regards to directions for future research (see Table 5).

**Table 4.** Critical Findings.

Review Finding Category	Review Findings
Critical findings regarding the results of the reviewed studies	• There is some evidence to suggest that providing information that challenges RMs can reduce jurors’ reliance on RMs during JDM. • Judicial directions were found to have some impact upon JDM; however, findings were mixed. • Expert witness testimony was found to have some effect on JDM; however, results were mixed. • Expert witness testimony may be more effective when presented after a complainant statement. • This review found no evidence that, under experimental conditions, a prosecution lawyer providing RM-debunking information to jurors reduces their use of RMs during JDM.
Critical findings regarding the critical appraisal of the reviewed studies	• Studies conducted to date are compromised by internal and external validity issues.

JDM = jury decision-making.

**Table 5.** Implications for Research, Practice, and Policy.

Type	Implication
Research	• Those conducting future research in this area should: ○ Conduct power analyses to ensure participant sample size is sufficient for the analysis undertaken. ○ Randomly allocate participants to conditions to address sample skew and bias between/within conditions. ○ Identify, measure, and control for potential confounding and extraneous variables inevitable in mock-trial designs. ○ Ensure no significant variation in mock-juror pre-trial RM beliefs, and other factors that could account for post-trial differences. ○ Use validated measurement scales to assess mock-juror case judgments alongside overall verdict decisions. For example, the Juror Decision Scale (Willmott et al., 2018) to assess complainant and defendant believability ratings. ○ Make use of contemporary RMA measure tools that are more subtle and reliable than their traditional counterparts. For example, the Acceptance of Modern Myths about Sexual Aggression scale (Gerger et al., 2007) or the Updated Illinois Rape Myth Acceptance Scale (McMahon & Farmer, 2011). ○ Recruit nationally or regionally representative (non-student) samples of jury-eligible participants to ensure findings can be generalized more widely. ○ Present live and/or video trial re-enactments, rather than vignettes to improve ecological and external validity. ○ Make use of genuine trial transcripts and employ expert panels of experienced CJS practitioners when designing simulations. ○ Incorporate group deliberations as a dependent variable given that mock-trial designs which ignore such a component detract from genuine jury-trial procedures and may amount to no more than pre-deliberation juror preferences.
Practice	• The following issues should be considered regarding the implementation of RM interventions: ○ How RM-debunking information should be presented (i.e., whether abstract or applied RM-debunking information is provided; whether RM-debunking information should be provided with or without explanations of the information—such as an explanation as to *why* reports are often delayed, rather than simply stating that reports are often delayed). ○ Who the intervention should be provided by (e.g., barristers, judges, expert witnesses, rape crisis counselors). ○ When the intervention should be presented (e.g., pre-trial, in-trial, post-trial, or at several timepoints).
Policy	• Given the findings of this review, and subject to the availability of research which addresses the recommendations made above, policy makers should give greater consideration to the benefits of providing some form of detailed RM education or debunking information to jurors, in-trial or pre-trial.

CJS = criminal justice system; RM = rape myths.

Judicial instructions were found to be somewhat effective, though not in all circumstances. Various proposals have been made as to how the effectiveness of judicial instructions may be advanced. Nitschke et al. (2023) found that judicial directions regarding complainant credibility led to participants becoming less influenced by stereotypes when evaluating complainants, but more skeptical of them overall. Given this, they suggested that instead of simply advising jurors not to depend upon unreliable credibility cues, jurors should be provided with alternative information as to how to evaluate credibility. This should be assessed in future research.

Expert testimony can have some effect on JDM (e.g., Ellison & Munro, 2009), though it is argued that the connection between such testimony and the case should be made explicit (Brekke & Borgida, 1988). However, Ellison and Munro (2009) argue that expert testimony does not have to be case specific. Rather they contend that general expert witness testimony may suffice. Ultimately, they posited that the reason why Brekke and Borgida (1988) found that case-specific testimony had more of an impact upon decision-making than did standard directions was that the latter presented facts alone, rather than facts followed by explanations of them. They suggest that general testimony can be effective if clear explanations of assertions are given. For example, rather than merely stating that reports are often delayed, testimony should provide explanations as to why this happens. They also suggest providing references to support stated facts. Future research should examine the impact of enacting such suggestions.

Ellison and Munro (2009) argued that given general expert witness testimony would refer only to claims that are fully supported by a consensus within the scientific literature, concerns regarding “battles of experts” are unfounded. Likewise, they dispute that introducing expert witnesses raises a danger of usurping the jury’s function. They note Vidmar and Schuller’s (1989) findings that jurors presented with expert testimony continued to utilize their own judgment, rather than deferring to the expert.

Ellison and Munro (2009) also propose that caution should be taken when considering Spanos et al.’s (1991) finding that cross-examination reversed the effect of expert testimony. They noted that mock-trial research exploring expert testimony in other types of trials has not found this effect of cross-examination. Spanos et al.’s (1991) study was also criticized for lacking realism as the cross-examination went beyond that considered acceptable in a real trial (Leverick, 2020) and participants did not deliberate. It would be beneficial to conduct more realistic research to examine the effects of cross-examination in the context of a rape trial.

Of the articles that assessed the effect of expert witnesses, one also examined the impact of complainants’ statements (Ryan & Westera, 2018), finding that presentation of both an expert witness’ statement and a complainants’ statement led to increased ratings of defendant blame and positively influenced deliberations. This suggests that complainants’ statements may bolster the impact of expert witnesses. Future research could explore this issue further.

The article that assessed the impact of the prosecution providing RM-countering information (Klement et al., 2019) found this provision ineffective. The authors suggested that information presented by individuals who are viewed as impartial by jurors may be more effective. They argued that, as jurors are aware of the adversarial nature of trials, they may be cautious of the information presented by the prosecution or expert witnesses, and may be more accepting of information presented by a judge or another party who they feel is impartial. Future research should compare the relative effectiveness of information presented by different individuals. In light of the proposal to implement a pre-trial video countering RM information for jurors in rape trials in England and Wales, research could compare videos that employ different speakers (e.g., judges, academic researchers) presenting the same information. In addition, qualitative research could be undertaken to explore how mock-jurors perceive the credulity of such RM-debunking information presented by different presenters.

Brekke and Borgida (1988) argue that the timing of the presentation of RM information is also important, reporting that expert witness testimony had greater impact when it was presented early in a trial. Provision of RM information at the outset of a trial might be the most beneficial, as doing so could prevent jurors from developing narratives based on RMs (Chalmers & Leverick, 2018; Henderson & Duncanson, 2016; Temkin, 2010). Nevertheless, it has also been suggested that a potential benefit of giving such directions during the summing up is that they may then be salient in jurors’ minds during deliberation (Ellison, 2019).

Given the arguments outlined regarding timing, Ellison (2019) suggested that the most effective approach may be to provide RM information to juries both pre- and post-trial. For example, judicial directions could be given at the outset of a trial, and then be reiterated by comments made by prosecutors or through repetition of such information via the judge’s summing up. Such an approach would concur with the primacy-recency effect (e.g., Murre & Dros, 2015), namely that individuals are more likely to retain new information that is presented at the beginning and the end of a learning period, rather than during the middle. Timing should be considered and explored in future research assessing different approaches to this issue.

Several recommendations are made to enhance the validity of future research. Researchers should conduct power analyses to ensure that samples are sufficiently large; randomly allocate participants to conditions; assess baseline similarity between groups; identify potential confounds and control for them in analyses; and use measures such as the Juror Decision Scale (Willmott, et al., 2018) to assess participants’ judgments of cases, which has been shown to have good composite reliability and differential predictive validity when assessing defendant and complainant believability.

An advantage of using such a scale is that it permits investigation of how jurors make decisions. The importance of exploring beyond verdicts and deliberations to examine decision-making processes is demonstrated by Spanos et al. (1991). They reported that although participants who viewed the cross-examination of an expert witness were more likely to give not-guilty verdicts than those who saw the testimony alone, there was no difference in participants’ belief in the complainant’s testimony across conditions. This demonstrates that the verdicts reached may not necessarily reflect participants’ belief in the complainant’s or the defendant’s testimony, or be solely based on such views. This suggests that including additional measures, such as believability scales, may provide a broader understanding of decision-making processes. The findings from future studies exploring these broader decision-making processes could, in turn, provide targets for educational interventions to assist JDM.

Future studies should use community samples of jury-eligible individuals to enhance the generalizability of findings to genuine court settings. Bornstein et al. (2017) argue that while this recommendation is questionable, given that results of mock-trial studies with student samples and samples from the general population are similar, influencing policy might be better achieved if the findings are drawn from studies with representative samples.

To enhance the ecological validity of mock-trials further, trial re-enactments rather than vignettes should be presented. Also, researchers should create case information and trial simulations based on real case transcripts, and employ expert panels to provide guidance.

Future research could also be improved with the routine inclusion of group deliberations. This is crucial to ensure high ecological validity, even where group deliberation is not the central focus of the planned analyses (Willmott et al., 2021). Ellison and Munro (2009) support this suggestion and have noted that while some researchers have utilized quantitative approaches in examining deliberations, often counting the number of references made to certain factors (e.g., Brekke & Borgida, 1988), they favored a contextual qualitative approach in their analysis. They argued that such an approach is required to explore the nature of deliberations with regards to the strength of views expressed and the dynamics of the juror interactions. Examining deliberations can also help to ascertain which factors influence verdicts (Spanos et al., 1991).

Finally, in the aforementioned discussion of internal validity, it was recommended that the JDS be used. Although this would enhance the understanding of JDM and would be an improvement on the use of unvalidated measures, careful consideration should be made with regards to the inclusion of this participant questionnaire. Use of the scale could reduce the external validity of the research, given that real jurors are not asked to provide such judgments. Thus, use of such a scale should depend on the aims of the research. The scale is appropriate when the study aims to understand decision-making, but less appropriate when attempting to recreate the real-world conditions of JDM. One way to incorporate the scale in the latter context would be to present the scale to participants in the post-deliberation phase of a study.

A strength of this review is the fact that a broad range of search terms was used, increasing the potential for relevant articles to be found and included. The rigor of the process was further enhanced via two of the co-authors reading the full text of a sample of articles screened by the first author to confirm the decisions made regarding inclusion and exclusion.

Arguably, this review is limited in that the included articles have a number of weaknesses. For example, the exclusion criteria did not dictate that articles be excluded on the basis of lack of randomization to conditions. Although it could be argued that the inclusion and exclusion criteria could have been more stringent, it can equally be said that it was crucial to consider all extant evidence, irrespective of quality (to a certain extent) given the lack of available research.

Finally, although the included articles were not assessed on the basis of existing critical appraisal tools, they were appraised based on criteria considered to be relevant to the types of studies reported on within them, which has been previously used by Dinos et al. (2015).

#### Summary

Reviewing the existing evidence base revealed mixed findings. The limited available evidence suggests that expert witness testimony, judicial instructions, and complainant statements may effectively impact upon JDM, such that reliance upon RMs is reduced. Nevertheless, assessment of such approaches produced mixed results across and within studies. Some studies did not report that the approaches assessed were effective, information regarding some of the myths did not affect decision-making, and where effects were reported, the information provided did not appear to impact upon all aspects of decision-making assessed. Further, it was reported that when RM information was presented by prosecutors, an impact upon decision-making was not observed; however, further research is required regarding this finding given that only one study assessed this approach. Beyond this, several further recommendations have been made regarding future research questions to explore, and with regards to the internal and external validity of such future studies. Given that research has consistently demonstrated that RMA can impact upon JDM in various ways, it is imperative that research in this area continues to determine how this issue may be best addressed.
